# Restructuring the FDA’s Food Program: Promises and Pitfalls

**DOI:** 10.3390/foods13203334

**Published:** 2024-10-21

**Authors:** Sammer Marzouk, Kranti C. Rumalla, Peter G. Lurie

**Affiliations:** 1 Department of Medicine, Northwestern Feinberg School of Medicine, Chicago, IL 60611, USA; sammer@northwestern.edu (S.M.); kranti.rumalla@northwestern.edu (K.C.R.); 2 Center for Science in the Public Interest, 1250 I Street NW, Suite 500, Washington, DC 20005, USA

**Keywords:** US Food and Drug Administration, reorganization, public policy, regulation, infant formula, Human Foods Program

## Abstract

Following an outbreak of Cronobacter sakazakii infection in infant formula, the US Food and Drug Administration commissioned a series of reports and then undertook a major reorganization of its food program. This article describes the changes that went into effect on 1 October 2024, why those changes are likely to improve the new Human Foods Program, and additional work that must be undertaken to enhance the agency’s impact upon public health.

The U.S. Food and Drug Administration (FDA) safeguards public health by regulating the nation’s food supply and ensuring the safety and nutritional value of the foods in the U.S. On 30 May 2024, the agency completed a major reorganization of its food program, which went into effect on 1 October 2024 [1]. The reorganization aims to enhance the agency’s ability to address emerging nutritional and food safety issues, streamline its own internal processes, and reimagine the agency’s inspection processes. In this article, we provide the historical background of the reorganization, the structure ultimately adopted by the FDA and its strengths and weaknesses, and the conditions necessary to maximize the new structure’s effectiveness.

Debates over the appropriate structure for US food programs date back decades, and have included proposals to unify the entire national food safety program, currently spread over more than a dozen agencies, under a single agency [2]. But the winds behind restructuring gained force with the 2021–2022 infant formula outbreak, in which infant formula contaminated with *Cronobacter sakazakii* may have caused four hospitalizations, including two deaths. All four infants had consumed formula produced at Abbott Nutrition’s facility in Sturgis, Michigan, prior to becoming ill [3]. The FDA’s investigation of the facility found *Cronobacter* bacteria (though not a genetic match to the outbreak strain) in high-risk areas of the production plant, along with other deficient practices [4], leading to a major recall and supply disruption, with worried parents scouring grocery shelves for months.

In response, FDA Commissioner Robert Califf first commissioned an internal agency review of the events. The report was completed in September 2022 and identified five major areas of need: modern information technology systems, sufficient staffing and training, updated emergency response systems, increased scientific understanding about *Cronobacter*, and assessment of the infant formula industry’s food safety practices and preparedness [5]. While the report provided recommendations in each of these areas, it failed to clearly outline the specific events surrounding the *Cronobacter* outbreak. Meanwhile, Congress mandated that the FDA ask the National Academies of Sciences, Engineering and Medicine to review issues related to the shortage. That report, issued on 25 July 2024, included a number of recommendations related to risk management planning, risk communication, and facilitation of formula imports [6].

Following the internal review, Commissioner Califf requested a report from the Reagan-Udall Foundation, an independent non-profit created by Congress to advance the mission of the FDA. The Foundation released its report on 6 December 2022, exposing the lack of organizational clarity within the FDA, including that there was a Deputy Commissioner for Food Policy and Response as well as a Director of the Center for Food Safety and Applied Nutrition (CFSAN), both of whom reported directly to the Commissioner [7]. The Foundation report provided five options for reorganizing the FDA’s human foods programs, but all sought to address this confusing structure (see Figure 1) and the historic de-emphasis at the FDA of food compared to medical products. The report also identified the need for cultural change within the food program and, like the earlier internal report, a need for human, financial, and information technology resources.

In the end, the agency opted for an approach that limited itself to FDA reform, eliminating both the Deputy Commissioner for Food Policy and Response and the Director of CFSAN, and instead created a new Deputy Commissioner for Human Foods, reporting directly to the FDA Commissioner, a more elevated status than that of any of the FDA’s other product centers (see Figure 2) [8]. This streamlines the organizational structure and gives the food program more prominence within an agency already managing multiple product lines.

Inevitably, the restructuring also touched on the Office of Regulatory Affairs (ORA), the FDA’s field office responsible for inspections, which has now acquired the more descriptive moniker Office of Inspections and Investigations (OII). The new Human Foods Program (HFP) now has authority over many of the OII’s food-related functions, including priority-setting and budgeting. Indeed, this reevaluation of the OII’s role will have ramifications throughout the agency, as each of the product centers gains greater authority over the OII’s budget and authorities in their respective areas; as a consequence, 1500 employees have been reassigned from the OII to the various product centers, including the HFP [9]. There were calls to subsume the food portions of the OII as well as the Center for Veterinary Medicine under a new deputy commissioner for foods, but the agency wisely elected a more limited, less disruptive approach that preserves the agency’s existing strengths.

The FDA’s restructuring plan also includes several key changes within the new HFP. A new office was established to focus on critical foods, such as infant formula and medical foods, with the goal of preventing future outbreaks and ensuring the safety and nutritional content of these essential products. The agency also has created a new Office of Integrated Food Safety System Partnerships to improve collaboration with state and local authorities, and a new Nutrition Center of Excellence, which aims to support the agency’s efforts in tackling diet-related chronic diseases and providing evidence-based nutrition guidance. Lastly, the FDA has established a new Office of Food Chemical Safety, Dietary Supplements, and Innovation, which consolidates the functions related to food additives and dietary supplements in a single office. This last proposal has engendered opposition from the supplement industry and Democratic senators alike, all fearing reduced visibility for dietary supplements [10]. The agency has stood firm.

While the restructuring of the FDA’s Human Foods Program is a step in the right direction, restructuring without resources will have limited benefit. Despite significant growth in its responsibilities, including the overhaul of the FDA’s food safety system through the Food Safety Modernization Act in 2011, the number of CFSAN employees has remained essentially flat for decades [11].

In conclusion, the restructuring of the FDA’s Human Foods Program is a necessary and overdue response to the structural issues within the FDA’s regulatory regime. By streamlining its organizational structure, improving collaboration with local authorities, and investing in critical areas such as nutrition and food safety, the FDA can better fulfill its mission of protecting and promoting the health of the American people. The success of the FDA’s restructuring efforts will depend on the availability of resources as well as its ability to adapt to the rapidly changing food environment, fostering an agency culture grounded in both innovation and food safety.

## Figures and Tables

**Figure 1:**
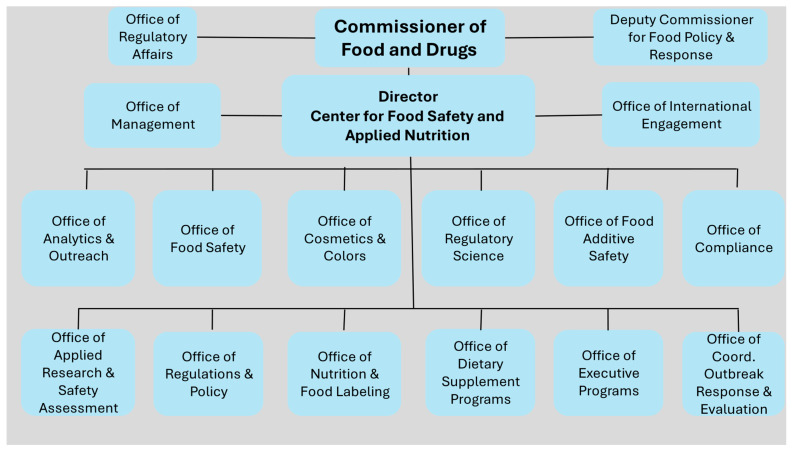
Previous Center for Food Safety and Applied Nutrition Organization Chart. Source: Adapted from: Center for Food Safety and Applied Nutrition Organizational Chart (June 2024). https://www.fda.gov/about-fda/fda-organization-charts/center-food-safety-and-applied-nutrition-organization-chart (downloaded 22 September 2024).

**Figure 2:**
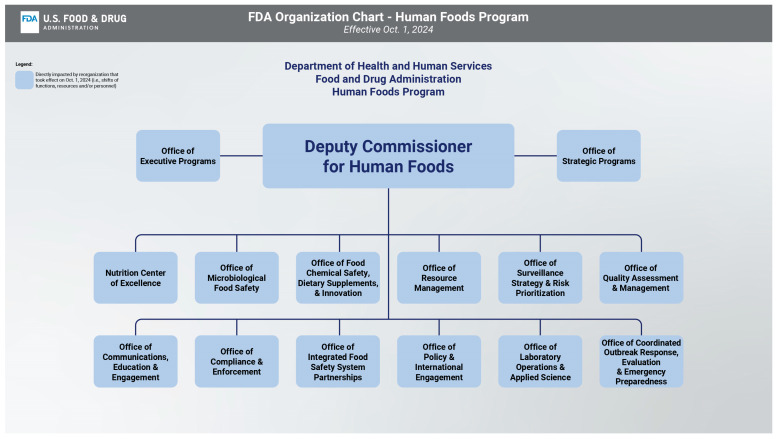
New Human Foods Program Organization Chart. Source: Approved Human Foods Program Organization Chart. https://www.fda.gov/media/172384/download (accessed on 1 July 2024).

## Data Availability

No new data were created or analyzed in this study. Data sharing is not applicable to this article.

## References

[1] Food and Drug Administration News Release. FDA’s Reorganization Approved for Establishing Unified Human Foods Program, New Model for Field Operations and Other Modernization Efforts|FDA. https://www.fda.gov/news-events/press-announcements/fdas-reorganization-approved-establishing-unified-human-foods-program-new-model-field-operations-and.

[2] Yeung B., Grabell M., Simon M. The Low-and-Slow Approach to Food Safety Reform Keeps Going Up in Smoke. https://www.propublica.org/article/the-low-and-slow-approach-to-food-safety-reform-keeps-going-up-in-smoke.

[3] Centers for Disease Control and Prevention Cronobacter Outbreak Linked to Powdered Infant Formula. https://www.cdc.gov/cronobacter/outbreaks/source-date/index.html.

[4] Food and Drug Administration Inspection of Abbott Laboratories Plant in Sturgis, MI (Form FDA 483), 18 March 2022. https://www.fda.gov/media/157073/download.

[5] Food and Drug Administration FDA Evaluation of Infant Formula Response. https://www.fda.gov/media/161689/download.

[6] Food and Nutrition Board Challenges in Supply, Market Competition, and Regulation of Infant Formula in the United States. https://nap.nationalacademies.org/catalog/27765/challenges-in-supply-market-competition-and-regulation-of-infant-formula-in-the-united-states.

[7] Reagan-Udall Foundation Operational Evaluation of the FDA Human Foods Program. https://reaganudall.org/sites/default/files/2022-12/Human%20Foods%20Program%20Independent%20Expert%20Panel%20Final%20Report%20120622.pdf.

[8] Department of Health and Human Services, Food and Drug Administration Statement of Organization, Functions, and Delegations of Authority. https://www.federalregister.gov/documents/2024/06/03/2024-11893/statement-of-organization-functions-and-delegations-of-authority.

[9] Al-Faruque F. FDA Leaders Detail Reorg Plans, Say 1500 ORA Staff Will Be Reassigned. https://www.raps.org/News-and-Articles/News-Articles/2024/1/FDA-leaders-detail-reorg-plans,-say-1,500-ORA-staf.

[10] Daniells S. Sens. Durbin & Blumenthal Express Concerns over Proposed Changes to FDA’s Supplement Office. https://www.nutraingredients-usa.com/Article/2023/08/26/Sens.-Durbin-Blumenthal-express-concerns-over-proposed-changes-to-FDA-s-Supplement-Office.

[11] Food and Drug Administration, Center for Food Safety and Applied Nutrition Presentation to Association of Food and Drug Officials, College Park, MD, 29 August 2024. https://www.cspinet.org/resource/safe-food-coalition-slides.

